# Individual component analysis of the multi-parametric cardiovascular magnetic resonance protocol in the CE-MARC trial

**DOI:** 10.1186/s12968-015-0169-2

**Published:** 2015-07-15

**Authors:** David P Ripley, Manish Motwani, Julia M. Brown, Jane Nixon, Colin C. Everett, Petra Bijsterveld, Neil Maredia, Sven Plein, John P. Greenwood

**Affiliations:** Multidisciplinary Cardiovascular Research Centre (MCRC) & Leeds Institute of Cardiovascular and Metabolic Medicine, University of Leeds, Leeds, UK; Clinical Trials Research Unit, University of Leeds, Clinical Trials Research House, 71-75 Clarendon Rd, Leeds, UK

**Keywords:** Magnetic resonance, Perfusion magnetic resonance imaging, Sensitivity, Specificity

## Abstract

**Background:**

The CE-MARC study assessed the diagnostic performance investigated the use of cardiovascular magnetic resonance (CMR) in patients with suspected coronary artery disease (CAD). The study used a multi-parametric CMR protocol assessing 4 components: i) left ventricular function; ii) myocardial perfusion; iii) viability (late gadolinium enhancement (LGE)) and iv) coronary magnetic resonance angiography (MRA). In this pre-specified CE-MARC sub-study we assessed the diagnostic accuracy of the individual CMR components and their combinations.

**Methods:**

All patients from the CE-MARC population (*n* = 752) were included using data from the original blinded-read. The four individual core components of the CMR protocol was determined separately and then in paired and triplet combinations. Results were then compared to the full multi-parametric protocol.

**Results:**

CMR and X-ray angiography results were available in 676 patients. The maximum sensitivity for the detection of significant CAD by CMR was achieved when all four components were used (86.5 %). Specificity of perfusion (91.8 %), function (93.7 %) and LGE (95.8 %) on its own was significantly better than specificity of the multi-parametric protocol (83.4 %) (all *P* < 0.0001) but with the penalty of decreased sensitivity (86.5 % vs. 76.9 %, 47.4 % and 40.8 % respectively). The full multi-parametric protocol was the optimum to rule-out significant CAD (Likelihood Ratio negative (LR-) 0.16) and the LGE component alone was the best to rue-in CAD (LR+ 9.81). Overall diagnostic accuracy was similar with the full multi-parametric protocol (85.9 %) compared to paired and triplet combinations. The use of coronary MRA within the full multi-parametric protocol had no additional diagnostic benefit compared to the perfusion/function/LGE combination (overall accuracy 84.6 % vs. 84.2 % (*P* = 0.5316); LR- 0.16 vs. 0.21; LR+ 5.21 vs. 5.77).

**Conclusions:**

From this pre-specified sub-analysis of the CE-MARC study, the full multi-parametric protocol had the highest sensitivity and was the optimal approach to rule-out significant CAD. The LGE component alone was the optimal rule-in strategy. Finally the inclusion of coronary MRA provided no additional benefit when compared to the combination of perfusion/function/LGE.

**Trial registration:**

Current Controlled Trials ISRCTN77246133

## Background

Coronary artery disease (CAD) is a leading cause of death and disability worldwide. Cardiovascular magnetic resonance (CMR) is recognised in international guidelines as a non-invasive imaging option for the investigation of suspected CAD [1–3]. The CE-MARC study was the largest prospective evaluation of the diagnostic accuracy of CMR in stable CAD to date [4, 5]. The trial adopted a multi-parametric CMR protocol assessing left ventricular (LV) function, myocardial perfusion, viability and coronary artery anatomy in a single study. A rigorous study design avoided referral bias by mandating that all patients underwent X-ray coronary angiography (XRA) as the reference test independent of the result of the CMR or single-photon emission computed tomography (SPECT) scans. The results from CE-MARC and its sub-analyses have shown that CMR had high diagnostic accuracy for suspected CAD in males and females, in single and multi-vessel disease, had higher overall diagnostic accuracy and was also cost effective compared to SPECT [6, 7].

Previous studies designed to determine the diagnostic accuracy of the individual components of the CMR examination have been small and revealed contrasting results. Some have shown the full multi-parametric approach had higher diagnostic accuracy over the individual components of the combined examination, although these were performed in selected populations [8–11]. Furthermore the clinical utility of imaging coronary artery anatomy for the detection of stenosis by magnetic resonance angiography (MRA) within already lengthy protocols remains to be established. Klein *et al.* demonstrated that MRA at 1.5 Tesla (T) did not add to the diagnostic accuracy over perfusion and late gadolinium enhancement (LGE) [11]. Other investigators have evaluated the effect of adding coronary MRA to stress perfusion and LGE on diagnostic performance in the intermediate to high risk group; when compared to invasive pressure-wire derived fractional flow reserve (FFR) at 1.5 T there was no significant improvement in diagnostic accuracy [12].

This predefined sub-study of CE-MARC compared the diagnostic accuracy of the full multi-parametric CMR protocol with the individual components, and their paired and triplet combinations. The aim was to determine the diagnostic accuracy of the individual components and their combinations in a large, prospective, real-world population of patients with suspected CAD requiring further investigation.

## Methods

### Study design

CE-MARC was a prospective study of 752 consecutive patients with suspected angina and at least one cardiovascular risk factor. Screening and recruitment occurred between March 2006 and August 2009 [4, 5]. All patients were scheduled to undergo SPECT and CMR (in randomized order), followed by XRA within 4 weeks. Inclusion and exclusion criteria have been previously published [4, 5]. Patients provided informed written consent and the study was approved by the local Research Ethics Committee and complied with the Declaration of Helsinki (2000).

All patients from the CE-MARC population were included in this pre-specified sub-analysis. CMR results were from the original, blinded visual read. The diagnostic accuracy of each individual core component of the multi-parametric CMR protocol (perfusion, LV function, MRA and LGE) was determined separately and then in paired or triplet combinations. The results were compared with the full multi-parametric protocol.

### CMR and analysis

The multi-parametric CMR (1.5-Tesla Intera CV, Philips, Best, The Netherlands) protocol and pulse sequence parameters have previously been described [4, 5]. The primary analysis used all four components of the multi-parametric CMR study. Criteria for a positive CMR result was any of the following: a) regional wall motion abnormality (RWMA) on cine imaging; b) hypoperfusion on stress/rest perfusion imaging; c) significant stenosis on MRA; d) infarct on LGE images (Table 1) following a ‘believe the positive rule’. Individual component image quality scores for CMR (cines, perfusion, LGE, MRA) were graded 1 (unusable) to 4 (excellent).

**Table 1:** Criteria for a positive CMR result in the CE-MARC study

Parameter	Method	Positive criteria
RWMA	Wall motion in each segment (17-segment model) was visually graded on post-stress cine imaging [0 = normal, 1 = mild-moderate hypokinesis, 2 = severe hypokinesis, 3 = akinesis, 4 = dyskinesis]	Wall motion Score ≥1 in two or more adjacent segments, or ≥2 in one or more segments
Ischemia	Perfusion in each segment (17-segment model)^a^ was visually graded at rest and then stress [0 = normal, 1 = equivocal, 2 = subendocardial defect, 3 = transmural defect, 4 = transmural defect and wall thinned]	Decrease in perfusion score ≥2 between rest and stress in any segment, or ≥1 in each of two adjacent segments^b^
Stenosis	Percentage of coronary artery luminal narrowing visually assessed on MRA	≥70 % stenosis or ≥50 % left main stem stenosis
Infarction	LGE images were visually assessed for hyper-enhancement in each segment (17-segment model) [0 = none, 1 = 1–25 %, 2 = 26–50 %, 3 = 51–75 %, 4= > 75 %]	Any score ≥1 in a pattern consistent with myocardial infarction

*RWMA* regional wall motion abnormality, *MRA* magnetic resonance coronary angiography, *LGE* late-gadolinium enhancement

^a^17-segment model excluding apical cap

^b^With the exception of change between ‘normal’ and ‘equivocal’, which was coded as ‘normal’

### X-ray angiography

XRA images were analysed by two experienced cardiologists blinded to the CMR and SPECT results. Significant CAD was defined as ≥70 % stenosis of a first order coronary artery measuring ≥2 mm in diameter, or left main stem stenosis ≥50 % by quantitative coronary angiography (QCA) (QCAPlus, Sanders Data Systems, Palo Alto, California, USA).

### Statistical analysis

Statistical analyses were performed by the Clinical Trials Research Unit, University of Leeds. Confidence intervals for the sensitivity, specificity, overall accuracy and positive (PPV) and negative predictive values (NPV) were calculated with the Wilson score method. Sensitivities and specificities were compared by the McNemar’s test, and predictive values were compared using the generalised score statistic. The positive (LR+) and negative likelihood ratios (LR-) were calculated using standard methods [13]. Assessment of the value of each component as “add on tests” were made with relative likelihood ratios [13]. Statistical analysis performed using with SAS software, version 9.2 at a two-sided 5 % significance level.

## Results

### Study population

Both CMR and XRA were available in 676 patients (mean 60 ± 9.5 years, 62 % male). For the individual components LGE was available in 674 (99.7 %), perfusion in 661 (97.8 %), ventricular function in 676 (100 %) and MRA in 597 (88.3 %). The prevalence of XRA defined significant CAD was 39 % and further demographic details are shown in Table 2.

**Table 2:** Summary of demographic and angiographic characteristics

		*n* = 676
Age (years)		60.3 ± 9.5
Male gender		421 (62 %)
Body Mass Index (kg/m^2^)		29.0 ± 4.3
Ethnicity	White	643 (95 %)
	Black	5 (1 %)
	Asian	24 (4 %)
	Other	4 (1 %)
Smoking status	Never smoked	236 (35 %)
	Ex-smoker	315 (47 %)
	Current smoker	125 (18 %)
Systolic Blood Pressure (mmHg)		138.1 ± 20.9
Diastolic Blood Pressure (mmHg)		79.0 ± 11.3
Previous admission for AMI or ACS		54 (8.0 %)
Previous PCI		37 (5 %)
Hypertension		347 (51 %)
Diabetes mellitus		85 (13 %)
	Type I	4 (5 %)
	Type II	81 (95 %)
Family history of premature CAD	Yes	392 (58 %)
	No	237 (35 %)
	Unknown	47 (7 %)
Total cholesterol (mmol/L)		5.2 (1.2)
Medication		
Aspirin and/or Clopidogrel		404 (60 %)
Statin		301 (45 %)
ACEi/A2 Receptor Blockers		229 (37.2 %)
Beta-blocker		203 (33.0 %)
**Patients undergoing X-ray angiography**		
Any significant stenosis		266 (39 %)
Triple Vessel Disease		40 (6 %)
Double Vessel Disease		83 (12 %)
Single Vessel Disease		143 (21 %)
LMS Disease		22 (3 %)
LAD Disease		169 (25 %)
LCx Disease		126 (19 %)
RCA Disease		105 (16 %)

Mean ± standard deviation. Number (percentage)

*AMI* acute myocardial infarction, *ACS* acute coronary syndrome, *PCI* percutaneous coronary intervention, *CAD* coronary artery disease, *ACEi* angiotensin converting enzyme inhibitor, *A2* angiotensin 2, *LMS* left main stem, *LAD* left anterior descending, *LCx* left circumflex, *RCA* right coronary artery

### Diagnostic accuracy

**Fig 1:**
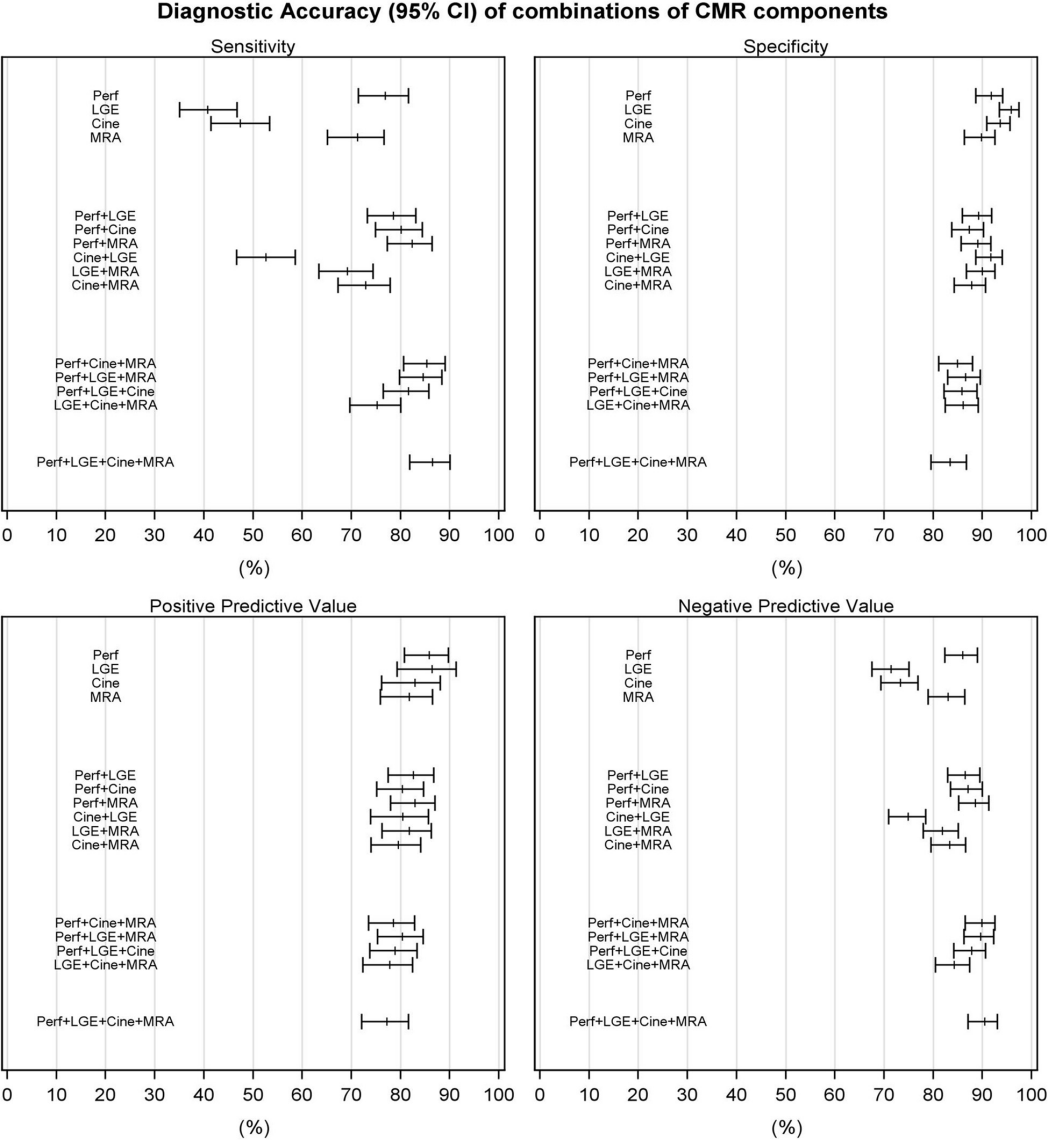
Diagnostic accuracy of the individual components and their combinations compared to the full multi-parametric CMR examination. Cine – Cine imaging; LGE – late gadolinium enhancement; Perf –perfusion imaging; MRA – magnetic resonance coronary angiography

The sensitivity of the combined CMR protocol was 86.5 % (95 % CI: 81.9–90.1), specificity 83.4 % (79.5–86.7), PPV 77.2 % (72.1–81.6 %), NPV 90.5 % (87.1–93.0) and overall diagnostic accuracy 84.6 % (81.7–87.1). The diagnostic accuracy of the individual components, paired and triplet combinations compared to the full multi-parametric protocol are presented in Table 3 and Fig. 1.

**Table 3:** Diagnostic accuracy of a multi-parametric CMR exam and its individual components, paired and triplet combinations compared to the reference test X-ray angiography

	Sensitivity (95 % CI)	Specificity (95 % CI)	PPV (95 % CI)	NPV (95 % CI)	Overall accuracy (95 % CI)
Overall multi-parametric CMR study (all components) (*n* = 676)	86.5 (81.8, 90.1)	83.4 (79.5, 86.7)	77.2 (72.1, 81.6)	90.5 (87.1, 93.0)	84.6 (81.7, 87.1)
**Individual CMR components**					
LGE (*n* = 674)	40.8 (35.0, 46.8)	95.8 (93.4, 97.4)	86.4 (79.3, 91.3)	71.4 (67.5, 75.0)	74.2 (70.7, 77.3)
Perfusion (*n* = 661)	76.9 (71.4, 81.6)	91.8 (88.7, 94.1)	85.8 (80.8, 89.7)	86.0 (82.4, 89.0)	85.9 (83.1, 88.4)
Ventricular function (*n* = 676)	47.4 (41.4, 53.4)	93.7 (90.9, 95.6)	82.9 (76.1, 88.1)	73.3 (69.3, 76.9)	75.4 (72.1, 78.5)
MRA (*n* = 597)	71.2 (65.1, 76.7)	89.8 (86.3, 92.5)	81.8 (75.9, 86.5)	83.0 (79.0, 86.4)	82.6 (79.3, 85.4)
**Paired combinations**					
Perfusion/LGE (*n* = 676)	78.6 (73.3, 83.1)	89.3 (85.9, 91.9)	82.6 (77.5, 86.8)	86.5 (82.9, 89.5)	85.1 (82.2, 87.5)
Perfusion/function (*n* = 676)	80.1 (74.9, 84.4)	87.3 (83.7, 90.2)	80.4 (75.2, 84.7)	87.1 (83.5, 90.0)	84.5 (81.5, 87.0)
Perfusion/MRA (*n* = 676)	82.3 (77.3, 86.4)	89.0 (85.6, 91.7)	83.0 (78.0, 87.0)	88.6 (85.2, 91.3)	86.4 (83.6, 88.8)
Function/LGE (*n* = 676)	52.6 (46.6, 58.6)	91.7 (88.6, 94.0)	80.5 (73.9, 85.7)	74.9 (70.9, 78.5)	76.3 (73.0, 79.4)
Function/MRA (*n* = 676)	72.9 (67.3, 77.9)	87.8 (84.3, 90.6)	79.5 (74.0, 84.1)	83.3 (79.5, 86.6)	82.0 (78.9, 84.7)
LGE/MRA (*n* = 676)	69.2 (63.4, 74.4)	90.0 (86.7, 92.5)	81.8 (76.2, 86.3)	81.8 (78.0, 85.1)	81.8 (78.7, 84.5)
**Triplet combinations**					
Perfusion/LGE/function (*n* = 676)	81.6 (76.5, 85.8)	85.9 (82.1, 88.9)	78.9 (73.7, 83.3)	87.8 (84.2, 90.6)	84.2 (81.2, 86.7)
Perfusion/LGE/MRA (*n* = 676)	84.6 (79.8, 88.4)	86.6 (82.9, 89.5)	80.4 (75.3, 84.6)	89.6 (86.3, 92.3)	85.8 (83.0, 88.2)
Perfusion/function/MRA (*n* = 676)	85.3 (80.6, 89.1)	84.9 (81.1, 88.0)	78.5 (73.5, 82.9)	89.9 (86.5, 92.5)	85.1 (82.2, 87.5)
LGE/function/MRA (*n* = 676)	75.2 (69.7, 80.0)	86.1 (82.4, 89.1)	77.8 (72.4, 82.5)	84.2 (80.5, 87.4)	81.8 (78.7, 84.5)

*CMR* cardiovascular magnetic resonance, *LGE* late gadolinium enhancement, *LR-* Likelihood Ratio Negative, *LR+* Likelihood Ratio Positive, *MRA* magnetic resonance coronary angiography

We have shown that of the individual components, perfusion had numerically the highest sensitivity (76.9 %), NPV (86.0 %) and overall diagnostic accuracy (85.9 %), whilst LGE had the highest specificity (95.8 %) and PPV (86.4 %) for the detection of significant CAD.

The maximum sensitivity (86.5 %) and NPV (90.5 %) for the detection of significant CAD by CMR was achieved when the full multi-parametric protocol was used, no individual component, paired or triplet combination outperformed the full multi-parametric protocol. However its lower specificity and PPV, meant that its overall diagnostic accuracy (84.6 %) was broadly similar to the majority of paired and triplet combinations (Table 3).

In terms of specificity, the individual components of perfusion (91.8 %), ventricular function (93.7 %) and LGE (95.8 %) all performed significantly better than the multi-parametric protocol (83.4 %) (*P* < 0.0001 for all). In addition, combining LGE with either ventricular function (91.7 %) or MRA (90.0 %) significantly improved the test specificity compared to the multi-parametric protocol (*P* < 0.0001 for each). For overall diagnostic performance, no individual component or combination was better statistically than the full multi-parametric protocol (Table 3). The use of coronary MRA had no additional diagnostic benefit in terms of overall diagnostic accuracy when performed within a multi-parametric protocol (84.6 % Vs. 84.2 %) (*X*^2^ = 0.3913,1df, *P* = 0.5316).

### The value of components as individual and add on tests: likelihood ratios

The highest likelihood ratio positive (LR+) was achieved when using LGE imaging alone (LR+ 9.81) signifying this individual component as the best approach for ruling in a diagnosis. All individual, paired and triplet combinations had higher LR+ than the full multi-parametric protocol (Table 4). However the full multi-parametric protocol had the lowest LR- (0.16) than all of the individual components and their combinations, signifying this as the best approach to rule out significant CAD. The absolute likelihood ratios for all of the components and their combinations are displayed in Table 4. Table 5 illustrates relative likelihood ratios using selected components as “add-on” tests to stress perfusion imaging alone, and the absolute number of new true and false positives cases produced with each combination.

**Table 4:** Likelihood ratios positive and negative for the multi-parametric CMR exam and its individual components, paired and triplet combinations compared to the reference test X-ray angiography

	Likelihood ratio + ve (95 % CI)	Likelihood ratio –ve (95 % CI)
Overall multi-parametric CMR study (all components) (*n* = 676)	5.21 (4.17, 6.51)	0.16 (0.12, 0.22)
**Individual CMR components**		
LGE (*n* = 674)	9.81 (6.02, 15.97)	0.62 (0.56, 0.68)
Perfusion (*n* = 661)	9.35 (6.70, 13.05)	0.25 (0.20, 0.31)
Ventricular function (*n* = 676)	7.47 (5.04, 11.07)	0.56 (0.50, 0.63)
MRA (*n* = 597)	7.01 (5.11, 9.61)	0.32 (0.26, 0.39)
**Paired combinations**		
Perfusion/LGE (*n* = 676)	7.32 (5.50, 9.75)	0.24 (0.19, 0.30)
Perfusion/function (*n* = 676)	6.31 (4.86, 8.20)	0.23 (0.18, 0.29)
Perfusion/MRA (*n* = 676)	7.50 (5.66, 9.94)	0.20 (0.15, 0.26)
Function/LGE (*n* = 676)	6.35 (4.51, 8.93)	0.52 (0.45, 0.59)
Function/MRA (*n* = 676)	5.98 (4.57, 7.83)	0.31 (0.25, 0.38)
LGE/MRA (*n* = 676)	6.92 (5.12, 9.35)	0.34 (0.29, 0.41)
**Triplet combinations**		
Perfusion/LGE/function (*n* = 676)	5.77 (4.51, 7.37)	0.21 (0.17, 0.28)
Perfusion/LGE/MRA (*n* = 676)	6.31 (4.90, 8.11)	0.18 (0.13, 0.24)
Perfusion/function/MRA (*n* = 676)	5.64 (4.46, 7.14)	0.17 (0.13, 0.23)
LGE/function/MRA (*n* = 676)	5.41 (4.21, 6.95)	0.29 (0.23, 0.36)

**Table 5:** Relative likelihood ratios and the numbers of new true positive and false positive cases produced by adding on further components sequentially to stress perfusion imaging in isolation

	Relative LR+	Relative LR-	New true positive cases produced	New false positives cases produced
Perfusion (+LGE)	0.78	0.91	7	11
Perfusion (+function)	0.68	0.89	9	18
Perfusion (+MRA)	0.79	0.76	16	12
Perfusion + LGE (+function)	0.79	0.89	8	14
Perfusion + LGE (+MRA)	0.86	0.74	16	11
Perfusion + function (+MRA)	0.89	0.76	14	10
Perfusion + function (+LGE)	0.91	0.94	4	6

*LGE* late gadolinium enhancement, *LR* likelihood ratio, *MRA* magnetic resonance coronary angiography

## Discussion

This pre-specified sub-study of the CE-MARC study has demonstrated the diagnostic accuracy of the individual components and the paired and triplet combinations from the multi-parametric CMR examination. The three main findings were that i) no individual component or combination of components outperformed the full multi-parametric protocol to rule out significant coronary artery disease; ii) the LGE component has the best performance to rule-in significant CAD; and iii) the addition of MRA to function/perfusion/LGE does not offer any incremental benefit.

### Likelihood ratios

We have shown the absolute likelihood ratio (LR) for each component and their combinations (Table 4) and demonstrated how many more (or less) times a particular component or combination result is likely in patients with CAD compared to those without the disease. LR is defined as the ratio of the expected test results in subjects with a certain disease to the subjects without disease, and they directly link the pre-test and post-test probability of the disease. A likelihood ratio of greater than 1 is associated with the presence of disease, whereas a ratio of less than 1 would indicate the test result is associated with the absence of disease. Importantly, as likelihood ratios are based on the ratio of sensitivity and specificity of an individual test, they are independent of disease prevalence, and can therefore be applied to different populations. The presented LRs can therefore be applied directly at the individual level and used to calculate how the probability of having CAD changes after the result of an individual component or combination of components of the CMR examination. Positive and negative likelihood ratios are therefore useful to understand the role of a test result in changing a clinician’s estimate of the probability of disease in a patient.

The LR for positive tests (LR+) is the likelihood that a given test result would be expected in a patient with the disease (i.e., how much more likely the positive test result is to occur in subjects with the disease compared to those without the disease). LR+ is the best indicator for a rule-in diagnosis and the higher the LR+ the more indicative of disease. LR+ is calculated as follows: LR+ = sensitivity/(1 – specificity). Therefore high sensitivity and specificity result in high LR+. The individual components of LGE (LR+ 9.81) and perfusion (9.35) had the highest LR+ amongst all the individual components and combinations with LGE benefitting from very high specificity to overcome poor sensitivity, and perfusion benefitting from both high sensitivity and specificity. For both components tested in isolation, a positive test finding increased the odds of the patient having CAD more than 9 fold. Therefore a positive LGE or perfusion test is a good test for ruling in the diagnosis of CAD.

Likelihood ratios for negative tests (LR-) demonstrate how much less likely the negative result will occur in subjects with the disease to the probability that the same result will occur without the disease. LR- is calculated as follows: LR- = (1 – specificity)/sensitivity and is a good indicator for ruling-out the diagnosis. For a single component, perfusion imaging produced the smallest likelihood ratio of disease for a negative finding (LR- 0.25): i.e., the odds of a patient having CAD were reduced by 75 % to one quarter of the pre-test odds with a normal perfusion result. By comparison, the odds of having CAD were only reduced by around 40 % with a negative LGE finding (LR- 0.62). Therefore for a single component, perfusion resulted in the greatest change in post-test odds of having coronary disease, and an overall diagnostic accuracy of 85.9 %. In terms of both positive and negative likelihood ratios, no paired or triplet combination offered a significant benefit over the best performing component of perfusion alone.

When combining the information from the four components in the full multi-parametric protocol using the “believe the positive” rule, the consequent reductions in specificity were not met by similar increases in sensitivity, which resulted in a comparatively low LR+ of 5.21. The full multi-parametric CMR examination, however, with all 4 components combined had the lowest LR- (0.16) indicating that the combination of all 4 components was best for ruling out CAD.

The high LR+, low LR- and high overall diagnostic accuracy of the single perfusion component demonstrates that perfusion imaging ought to have most influence on a physician's risk stratification of the patients’ likelihood of having significant underlying CAD. We have therefore shown the relative likelihood ratios of the perfusion component as the starting point, and building on this using selected combinations as “add on” tests, highlighting the number of new true and false positive cases produced by each combination (Table 5). This analysis showed that no add on test to perfusion imaging is preferable for ruling in the diagnosis (since all add on tests reduce the relative LR+), but adding on components can improve the rule-out value of the CMR examination (all add on tests reduce the LR-).

### Comparative literature

There have been a number of other studies analysing the diagnostic performance of the components of the CMR examination, although none of this magnitude and many of which being performed in highly selected populations.

One study analysed the diagnostic accuracy of CMR components in 100 patients preselected for X-ray coronary angiography (≥70 % stenosis as the reference standard) [8]. The CMR protocol included wall motion, stress and rest perfusion and LGE. The analysis algorithm considered LGE images first with presence of severe CAD diagnosed if LGE was positive in an ischaemic pattern. If LGE was negative the perfusion images were analysed and a reversible defect used to diagnose CAD. This analysis algorithm had a sensitivity (89 %) and specificity (87 %) - which was similar to the CE-MARC study. In terms of individual components compared to CE-MARC, the perfusion component in this previous study had the highest sensitivity (84 % vs. 77 % in our population) although with a significantly lower specificity (58 % vs. 92 %). Wall motion scoring was not considered in their analysis algorithm; cine images were acquired and had a similar sensitivity (49 % vs. 47 %) but lower specificity (73 % vs. 94 %) than in our study.

In patients with non ST-segment elevation myocardial infarction our group has previously evaluated the diagnostic accuracy of all 4 components of the CMR examination, performed within 72 h of presentation, with an overall sensitivity of 96 %, specificity 83 %, PPV 96 % and NPV 83 % [9]. Once again the perfusion component of the examination yielded the highest sensitivity (88 %), although in this study it was higher than when compared to our stable elective population (77 %).

Cury *et al.* studied a mixed cohort of 47 patients (14 with previous MI) and also demonstrated that stress perfusion imaging had the highest sensitivity (81 %) and LGE the highest specificity (94 %) [10]. The maximum diagnostic accuracy was achieved with the combination of stress perfusion and LGE, and unsurprisingly this was again higher in the sub-group of patients with previous myocardial infarction than those with suspected CAD and no prior infarction (93 % vs. 86 %).

The clinical utility of imaging coronary artery anatomy with dedicated coronary MRA protocols in expert centres has been demonstrated to have good diagnostic accuracy for the detection of proximal CAD [14]. Technical advances at 3.0 Tesla and using a 32 channel coil have been shown to further improve signal to noise ratio and overall accuracy compared with initial reports, yielding sensitivities of 92-96 % [15, 16]. However, the efficacy of coronary imaging within a combined CMR protocol remains to be established. Klein *et al.* performed coronary MRA, stress and rest perfusion and LGE imaging on 54 patients with suspected CAD, again showing the perfusion component was the most accurate alone (sensitivity 87 %, specificity 88 %). They showed that the addition of LGE to stress perfusion imaging did not improve the overall diagnostic accuracy (sensitivity 88 %, specificity 88 %). In terms of coronary imaging, 15 % of overall MRA had non-diagnostic image quality; whole heart MRA had significantly inferior diagnostic accuracy due to poor specificity (sensitivity 92 %, specificity 56 %) unless only those with excellent MRA image quality (*n* = 18, 33 %) were analysed, whereupon it remained similar to the perfusion component alone (sensitivity 86 %, specificity 91 %) [11]. Other investigators have evaluated the effect of adding coronary MRA to stress perfusion CMR on diagnostic performance; when compared to invasive pressure-wire derived fractional flow reserve (FFR) at 1.5 T there was no significant improvement in diagnostic accuracy [12].

Coronary MRA remains a time consuming acquisition, which often is non-diagnostic when performed within an already long multi-parametric protocol. In our study 79 patients (11.7 %) had non-diagnostic coronary MRA images. Furthermore, in those with adequate or excellent image quality (*n* = 597), the addition of the coronary MRA made no difference statistically on the overall diagnostic accuracy of the CMR examination. Equally, whilst some triplet combinations with MRA offer similar diagnostic accuracy, the components of cine, LGE and perfusion imaging offer clinical information above and beyond detection of coronary disease (i.e., left ventricular volumes/ejection fraction, myocardial viability and ischaemic burden) which may have additional prognostic importance.

## Conclusions

From this pre-specified sub-analysis of the CE-MARC study, using the original blinded visual-read, we have demonstrated the diagnostic accuracy of the individual components and their combinations from the full multi-parametric CMR exam. In patients presenting with stable chest pain, the stress perfusion component of the multi-parametric CMR exam was the single most important component for overall diagnostic accuracy. However, the full combined multi-parametric protocol was the optimal approach for disease rule-out, and the LGE component best for rule-in. The inclusion of coronary MRA had no additional overall diagnostic benefit within a multi-parametric protocol.

## Abbreviations

CAD: Coronary Artery Disease
CMR: Cardiovascular Magnetic Resonance
FFR: Fractional Flow Reserve
LGE: Late Gadolinium Enhancement
LR: Likelihood Ratio
LR+: Likelihood Ratio for Positive Tests
LR-: Likelihood Ratio for Negative Tests
LV: Left Ventricle
MRA: Magnetic Resonance Angiography
NPV: Negative Predictive Value
PPV: Positive Predictive Value
RWMA: Regional Wall Motion Abnormality
SPECT: Single Photon Emission Computed Tomography
XRA: X-Ray Angiography
